# Expanding the repertoire of miRNAs and miRNA-offset RNAs expressed in multiple myeloma by small RNA deep sequencing

**DOI:** 10.1038/s41408-019-0184-x

**Published:** 2019-02-19

**Authors:** Luca Agnelli, Andrea Bisognin, Katia Todoerti, Martina Manzoni, Elisa Taiana, Serena Galletti, Giovanna Cutrona, Enrico Gaffo, Stefania Bortoluzzi, Antonino Neri

**Affiliations:** 1 Department of Oncology and Hemato-Oncology, University of Milan, Milan, Italy; 2 Department of Molecular Medicine, University of Padua, Padua, Italy; 3 Hematology 1, Fondazione IRCCS Ca’ Granda Ospedale Maggiore Policlinico, Milan, Italy; 4 Molecular Pathology Unit, IRCCS Ospedale Policlinico San Martino, Genova, Italy

## Abstract

Microarray analysis of the multiple myeloma (MM) miRNome has unraveled the differential expression of miRNAs in cytogenetic subgroups, their involvement in the tumor biology and their effectiveness in prognostic models. Herein, the small RNA transcriptional landscape in MM has been investigated exploiting the possibilities offered by small RNA-seq, including accurate quantification of known mature species, discovery and characterization of isomiRs, and miRNA-offset RNAs (moRNAs). Matched small RNA-seq and miRNA GeneChip^®^ microarray expression profiles were obtained in a representative panel of 30 primary MM tumors, fully characterized for genomic aberrations and mutations. RNA-seq and microarray gave concordant estimations of known species. Enhanced analysis of RNA-seq data with the *miR&moRe* pipeline led to the characterization of 655 known and 17 new mature miRNAs and of 74 moRNAs expressed in the considered cohort, 5 of which (moR-150-3p, moR-24-2-5p, moR-421-5p, moR-21-5p, and moR-6724-5p) at high level. Ectopic expression of miR-135a-3p in t(4;14) patients, upregulation of moR-150-3p and moR-21-5p in t(14;16)/t(14;20) samples, and of moR-6724-1-5p in patients overexpressing CCND1 were uncovered and validated by qRT-PCR. Overall, RNA-seq offered a more complete overview of small non-coding RNA in MM tumors, indicating specific moRNAs that demand further investigations to explore their role in MM biology.

## Introduction

Many studies highlighted the relevant role of microRNAs (miRNAs) in the pathology of multiple myeloma (MM)^1^. Microarray and quantitative real-time PCR (qRT-PCR) analyses of the miRNome of plasma cells (PCs) from primary MM tumors have unraveled the differential expression of miRNAs and miRNA clusters in specific molecular subgroups, exemplarily the marked upregulation of the *miR-99b/let-7e/miR-125a-5p* cluster in t(4;14)^2–4^. We and others have demonstrated the effectiveness of miRNA expression to predict outcome and proficiently integrate ISS-based models^5,6^. The prognostic role of miRNAs in the form of circulating exosomal species is also emerging^7,8^. Recently, taking advantage of proprietary and publicly available transcriptional data, including paired miRNA and gene expression profiles of primary tumors, we provided a global and integrated view of miRNA transcriptional relationships in MM^9,10^.

However, to date, the investigation of miRNA expression in MM has been limited to microarray or PCR approaches. Albeit the cohorts and the number of investigated RNAs became increasingly extensive, the study of small RNA has still not exploited all the possibilities offered by next-generation sequencing. These include, but are not limited to, (i) improved detection of low-expressed mature species, (ii) accurate quantification of isomiRs^11^, and (iii) comprehensive investigation of miRNA-offset RNAs (moRNAs)^12,13^. Recent studies have in fact demonstrated that almost half of the identified miRNA genes encode up to four stable small RNA species: two miRNAs and two moRNAs, which are likely to be produced by the RNAse III-like processing of pre-miRNA hairpins and stem from the sequences immediately adjacent to the two mature −5p and −3p miRNAs^13–15^. Like miRNAs, moRNAs are ~20 nt long and are regulated at different developmental stages^12,16^. Langenberger et al. firstly reported that many human pre-miRNAs are processed to produce moRNAs in a systematic way. We and others have uncovered moRNA expression in pigs, among which it is worth reporting that of moR-21-5p, stemming from the processing of extended precursor hairpins^17,18^. Very recently, the transcriptional pattern of short non-coding RNAs, including moRNAs, has been characterized by deep sequencing in normal and neoplastic CD34+ cells, evidencing the specific and miRNA-independent modulation of moRNAs derived by non-canonically processed hairpins^19^. Several evidence, ranging from observational studies that reported high-sequence conservation between human and other species^20^ to recent functional investigations in vitro^21,22^, concordantly indicate that moRNAs could be functional RNAs. Based on these premises, the aim of the present work is to provide a more complete view of miRNA transcriptional landscape in MM, through RNA-sequencing (RNA-seq) analysis in a panel of primary tumors representative of the major molecular types.

## Methods

### Patients

Bone marrow aspirates were obtained during standard diagnostic procedures from 30 MM at diagnosis. PCs were purified using CD138 immunomagnetic microbeads and the purity of the positively selected PCs (>90%) was assessed using flow cytometry as previously reported^23,24^. All PC dyscrasia cases were investigated by fluorescence in-situ hybridization for the major immunoglobulin heavy chain locus (IGH@) translocations and genetic lesions, and MM patients were stratified according to molecular prognostic stratification^25^ (Supplementary Table 1). Mutational status of *BRAF*, *NRAS* and *KRAS*, *DIS3*, *FAM46C*, and *TP53* genes were assessed by targeted sequencing from genomic DNA by the Genome Sequencer Junior instrument (Roche-454 Life Sciences, Penzberg, Germany) as previously described^26–28^ (Supplementary Table 1). Written informed consent was obtained from all patients in accordance with the Declaration of Helsinki. The study was approved by the Ethical Committee of the University of Milan (N° 24/15, May 06 2015).

### Small RNA-sequencing

RNA-seq data were generated at the next-generation sequencing service of Parco Tecnologico Padano (Lodi, Italy). Small RNA cDNA libraries (for transcripts < 30 nt) were built following standard Illumina Truseq^®^ Small RNA protocol, starting from 2 μg of total RNA for each sample. BluePippin system was used for fragments selection within the range of 145–160 bp. RNA-seq was performed on Illumina HiSeq platform. A 50-cycle single-reads sequencing was run to generate the tags corresponding to small-RNAs.

### Small RNA detection, discovery, and quantification

Small RNA data analysis was conducted with an extended version of the *miR&moRe* pipeline (Supplementary Figure 1)^29^ (detailed procedure as described in Gaffo et al.^18^). Briefly, the first step was reads preprocessing for adapter removal, namely discarding “adapter-only” or unclipped reads. Then, reads with a length range between 15 and 30 nt (which encompassed the length range of human annotated miRNAs in miRBase and possible moRNAs and/or shorter/longer isomiRs) were considered for the further quality-based filtering, which kept reads with mean base quality higher than 30 and at most 2 nucleotides with quality under 20. Reads belonging to unique sequences with less than 10 reads counts each were discarded to remove ground noise. Then, filtered reads have been mapped, using Bowtie v1.1.0^30^, both to the GRCh38 genome assembly and the known hairpins sequences (miRBase version 21^31,32^) extended in either directions by additional 30 bp. Reads mapping to more than five different loci on the genome out of miRBase annotated hairpins have been discarded to reduce noise.

The following steps implemented in *miR&moRe* pipeline are then performed: (i) quantification of known miRNAs and (ii) isomiR characterization; in parallel, (iii) discovery of sister miRNAs and (iv) possible miRNA-offset RNAs produced from the considered hairpin precursors^18^. Known mature miRNAs are first recognized, then for hairpins containing only one annotated mature miRNA, RNAfold is used to define the most probable hairpin structure and the region complementary to the known miRNA, thus identifying the most probable miRNA duplex, reaching the identification of sister miRNAs. Expressed small-RNAs with the central nucleotide localized upstream of the region covered by the 5′ mature miRNA or downstream of the region covered by the 3′ mature miRNA, were considered 5′-moRNAs and 3′-moRNAs, respectively. The pipeline outputs known miRNA read counts, detects sequence and quantification data for new miRNAs and moRNAs, and lists of variants (isomiRs) for all the small-RNAs found in each sample, with quantification of each isomiRs. Then, the merged matrix of raw read counts in all considered samples underwent normalization using the R/Bioconductor package *DESeq*.

Cluster analysis of expression profiles was performed using standard *hclust* methods in R. Differential expression tests were conducted using the Bioconductor Package *edgeR*^33^; negative binomial generalized log-linear model was fitted to the reads count for each short RNA and then exact test *p*-values were calculated for every contrast of interest (each group, individually, vs the others, as a whole). Multiple test correction was performed according to Benjamini–Hochberg method.

### MiRNA array expression profiling

Total RNA samples from the same 30 MM patients were profiled on the GeneChip® Human miRNA v3.0 array (Affymetrix, Santa Clara, CA, USA). Normalized expression data of 1768 human mature miRNAs were extracted using RMA normalization^3^ and custom annotations based on miRBase release 21 (http://brainarray.mbni.med.umich.edu/Brainarray/Database/CustomCDF/21.0.0/mirbasef.asp).

### moRNA-target prediction

The identification of putative moRNA binding sites and corresponding heteroduplexes on mRNA target sequences was run using the downloadable dynamic tool of RNA22^34^ prediction algorithm (https://cm.jefferson.edu/rna22/), under customized parameters (i.e., default setting on sensitivity/specificity ratio, 7 nucleotides on seed region without any unpaired base allowed, one G:U wobble accepted, 14 as minimum required number of base-pairs between the moRNA and the target, −20 kcal/mol maximum allowed free energy). A list of 764 genes mapped to GRCh38 primary assembly and associated with MM (from now on, referred as “MM-genes”) was downloaded from NCBI database (https://www.ncbi.nlm.nih.gov/gene) and a total of 17,356 fasta-formatted sequences for all the MM-genes annotated trasncripts was used for the downstream analysis.

### Quantitative real-time PCR (qRT-PCR) validation

Selected mature miRNAs underwent qRT-PCR validation using custom TaqMan™ small RNA assays (Applied Biosystems) in accordance with the manufacturer’s protocol. Specifically, commercial assay was purchased for miR-135a-5p, whereas made-to-order TaqMan™ assays were designed on the most represented sequences for moR-21-5p (CTCCATGGCTGTACCACCTTGTCGG), moR-150-3p (GGGACCTGGGGACCCCGGCACCGGCAGG), and moR-6724-1-5p (TGTGGGGGAGAGGCTGTCGCTGCGCTTCTGGGCC). All the assays were normalized on the basis of the RNU48 TaqMan™ miRNA Assays-Control, used as housekeeping in view of its abundance and very low variability within PCs. Relative miRNA expression was quantified using the 2^−∆Ct^ method (Applied Biosystems, User Bulletin No.2). As a normal counterpart for moRNA expression evaluation, a representative panel of normal hemopoietic tissue samples available in our Institution biobank were used: 4 PCs, 5 germinal center B-cells, 5 naive B-cells, and 5 memory B-cells samples from tonsil avulsion during standard surgery, together with 4 mononucleated cells samples derived from peripheral blood (PBMC) of healthy donors, obtained as previously described^35^.

## Results

To provide an exhaustive view of the small-RNAs in MM, combined small RNA-seq (sRNA-seq) data and array expression profiles of a cohort of 30 primary MM tumors were considered. The selected MM cases (Supplementary Table 1) are representative of common molecular features in MM: IGH translocation with prognostic relevance (4p16, *7* cases; MAF, 4 cases*;* and 11q13 groups, 8 cases)^6^; 16 cases had 1q gain, 18 del(13) and 3 del(17); 8 cases were hyperdiploid (the number of cases with each molecular feature was chosen to optimize discovery power and costs).

### Deep characterization of small RNA in myeloma

Small RNA-seq data were generated in a cohort of 30 MM samples at diagnosis. Overall, 18.7 million average sRNA-seq reads per sample were obtained and 90.3% on average passed filtering steps (Supplementary Figure 2). Analysis of RNA-seq data by *miR&moRe* led to the quantification and molecular characterization of known miRNA species, integrated with the definition of isomiR sequences, and to the de novo identification of miRNA and moRNAs.

### Known and new miRNAs

A total of 672 expressed miRNAs were detected in at least one sample, including 655 known and 17 newly annotated miRNAs.

**Fig. 1:**
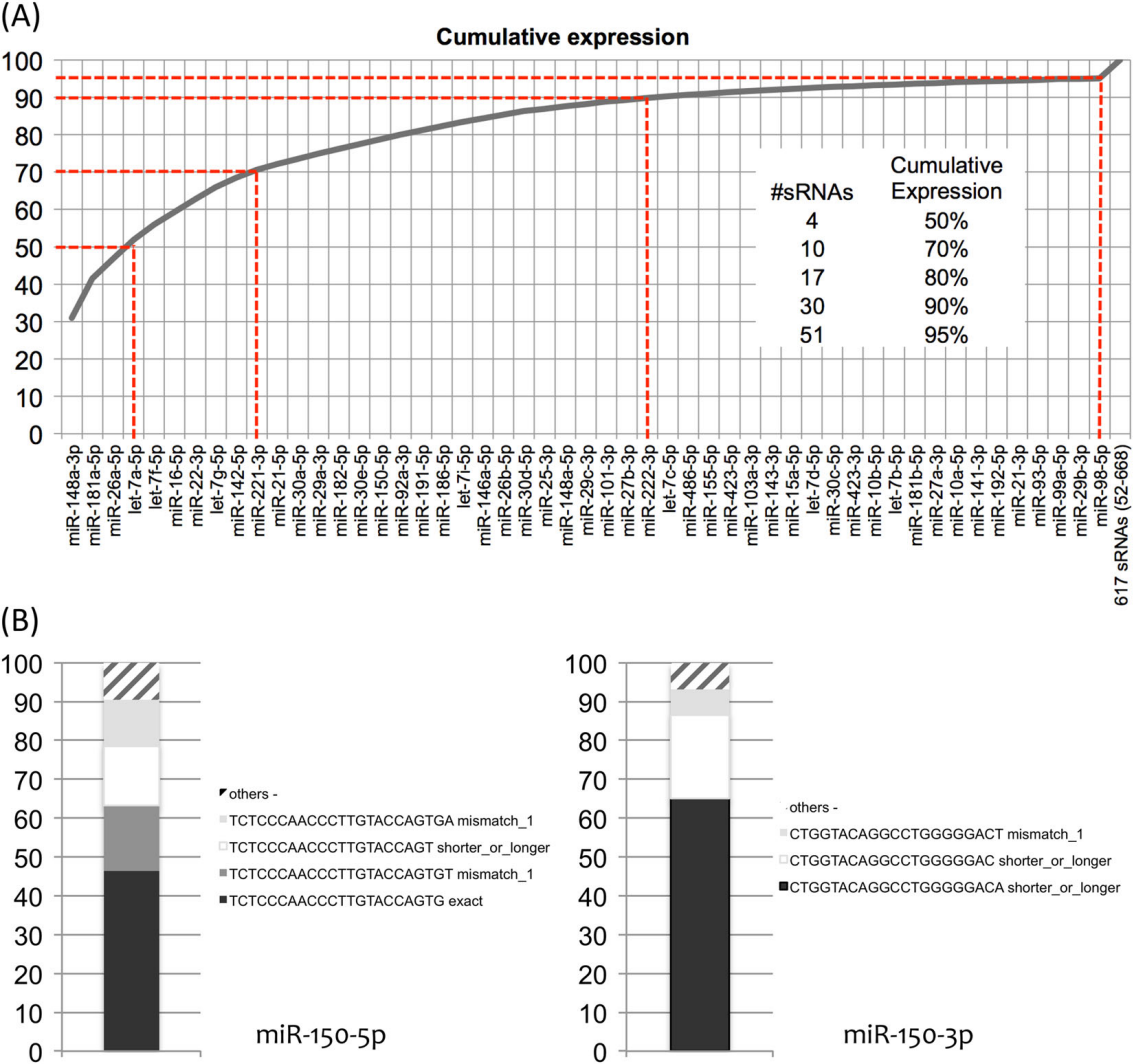
MiRNA expression in MM according to small RNA-seq: **a** Cumulative expression of sRNAs, ordered by expression level; **b** percentage of different isomiR species for miR-150-5p and -3p

Notably, only 51 known miRNAs (i.e. <2% of the 2813 annotated mature miRNAs in miRbase v21) represented up to 95% of the observed expression signals in MM, with a few of them very highly expressed: namely, the sum of the reads of only four mature miRNAs (miR-148a-3p, miR-181a-5p, miR-26a-5p, let-7a-5p) represented up to 50% of the expressed transcripts (Fig. 1a). Our analysis led also to the identification of 17 still undescribed, mature miRNAs originating from the annotated stem-loops. These new species were in general weakly expressed, but 6 new miRNAs had average expression over the median of all detected sRNAs (Supplementary Table 2).

The signal of each miRNA was calculated as the sum of the contribution of “exact” (i.e., with sequence and boundaries according to miRbase annotation) together with isomiR variants, namely those exceeding the known miRNA boundaries for at most three nucleotides, likely resulting from alternative Dicer processing or post-processing modifications as described^12^, or showing mismatches (at most one anywhere or two in the 3′ half of the sequence), compatible with possible SNPs or edited bases^18^. Overall, exact variants contributed only to 40.7% of total normalized read counts. Of the remaining 59.3%, the majority of isomiRs (76.4%) was represented by shorter or longer mature species without mismatches. Importantly, as previously reported, not all the miRNA species had the exact sequence (i.e., the mature miRNA annotated in miRbase) represented among detected isomiRs. In other words, the exact sequence may not necessarily represent the most abundant isomiR, as well as in some extreme cases “non-exact” isomiR contribution may represent up to the whole signal of the mature miRNA. Figure 1b reports the exemplar case of miR-150, where the −3p miRNA is entirely represented by mismatched or shorter/longer sequences.

Matched miRNA expression profiles obtained in the same sample set with RNA-seq and with microarray technologies were compared. Very good concordance is observed for higher signals, despite an apparently quite poor correlation (*r* = 0.24) when all the expressed species were considered. Both the scatter plot (Supplementary Figure 3A) and the array expression-based stratification of the correlation coefficients of each transcript (Supplementary Figure 3B) clearly indicated that any dissimilarity depended mostly on array expression signals just above the noise level.

### moRNAs

In the profiled series of 30 MM cases, 74 moRNAs were identified. Notably, several moRNAs had high and group-specific expression, as detailed below.

Overall, moRNAs represent around 10% of expressed sRNAs species and show prevalently a low abundance, accounting globally for <1% of detected sRNA expression, concordantly with previously reported evidence^21^. Nevertheless, 25 moRNAs had expression over the median of the sums of reads across all the samples, and 5 of them in the upper quartile of most expressed sRNAs (Table 1).

**Table 1:** Five moRNAs presenting highest expression across the dataset

moRNA	Precursor miRNA	Sequence	Rank
moR-150-3p	mir-150	GGGACCTGGGGACCCCGGCACCGGCAGG	148
moR-24-2-5p	mir-24-2	TGGCCTCCCTGGGCTCTGCCTCC	149
moR-421-5p	mir-421	TAATCCGGTGCACATTGTAGG	163
moR-21-5p	mir-21	CTCCATGGCTGTACCACCTTGTCGG	164
moR-6724-5p	mir-6724-1	TGTGGGGGAGAGGCTGTCGCTGCGCTTCTGGGCC	191
	mir-6724-2		
	mir-6724-3		
	mir-6724-4		

For each moRNA, the precursor miRNAs, the sequence and their ranking among the 768 sRNAs detected and ordered according to the sum of reads across the 30 myeloma samples are indicated

### sRNA-specific expression characterizes MM molecular subgroups

Differential expression analysis of expression profiles based on RNA-seq data identified miRNAs and moRNAs with expression specific/variable in each the 4p16, MAF, 11q13, and CCND1+ group.

The analysis of differentially expressed miRNA between MM prognostic subgroups indicated good level of overall concordance between the results obtained using microarray and RNA-seq approaches: more than 90% of the differentially expressed miRNAs resulting from microarray analysis were concordantly detected by RNA-seq. Conversely, as expected, only 80% of differentially expressed transcripts detected by RNA-seq were also identified by microarray analysis, in all likelihood in virtue of lower noise and the detection unconstrained to pre-determined sequences offered by RNA-seq (Supplementary Table 3).

**Fig. 2:**
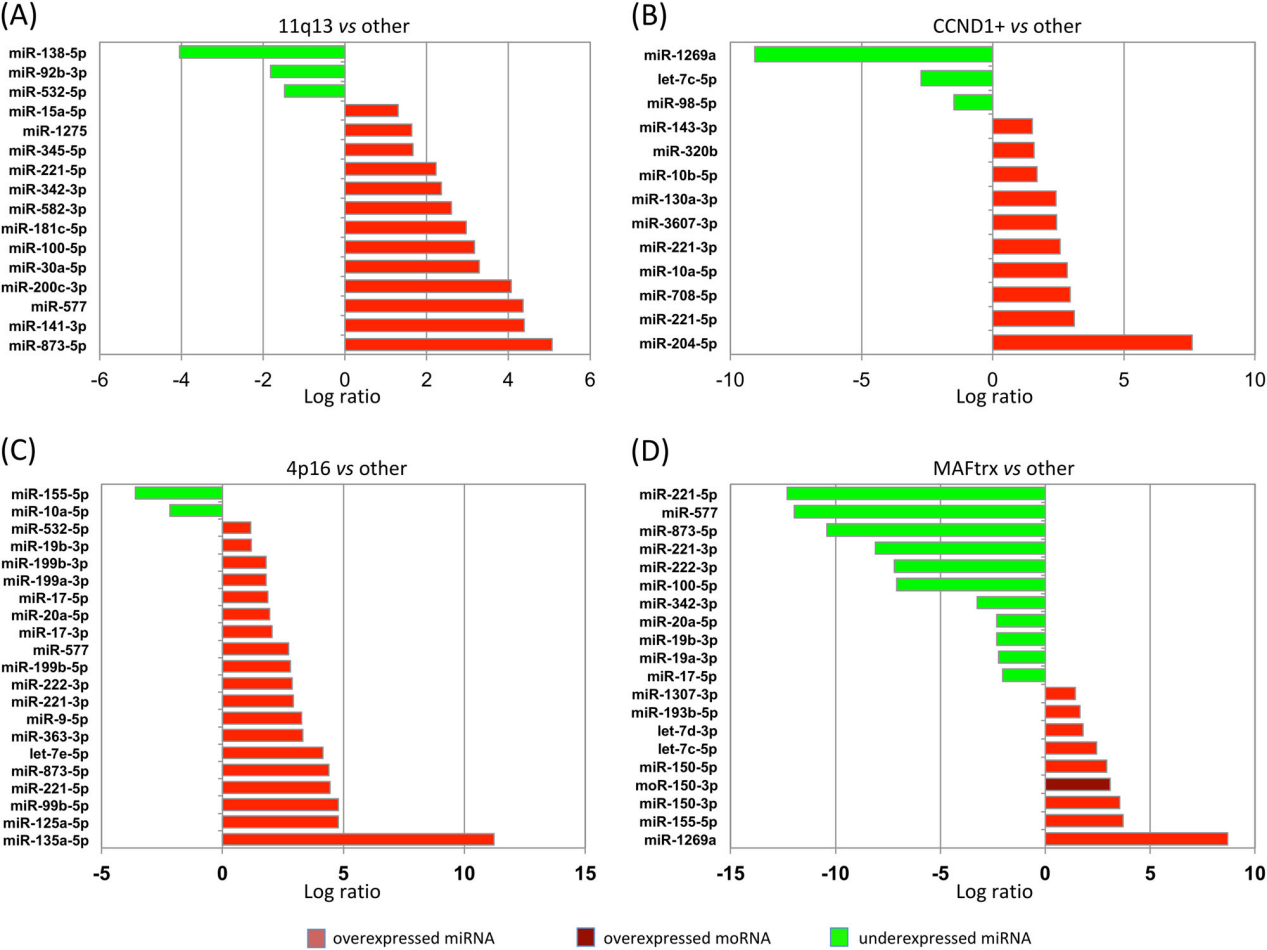
MiRNAs and moRNAs differentially expressed between MM prognostic subgroups. Histograms of relative fold changes of the differentially expressed miRNA, among those in the upper quartile of average expression, in **a** 11q13, **b** CCND1+, **c** 4p16, and **d** MAF genes translocated (MAFtrx) patients. Green indicates downregulation and red indicates upregulation (moRNA is shown in dark red)

Figure 2 shows the differentially expressed transcripts whose sum of reads exceeded the third quartile of all the sums of reads. Marked upregulation of the well-described miR-99b/let-7e/miR-125a-5p cluster was detected in t(4;14) patients by RNA-seq and arrays. Of interest, abnormal and specific expression of miR-135a-5p in t(4;14) patients was unraveled by RNA-seq, most likely overcoming hybridization biases due to sequence similarity within miR-135 family that might affect microarray investigation and thus previous reports^3^.

MiR-135a-3p, originating from the same stem loop of miR-135a-5p but expressed at relatively weaker levels, showed slight but anyway specific upregulation in t(4;14) patients, in line with the notion of imbalance in the abundance representation of sister mature species.

**Fig. 3:**
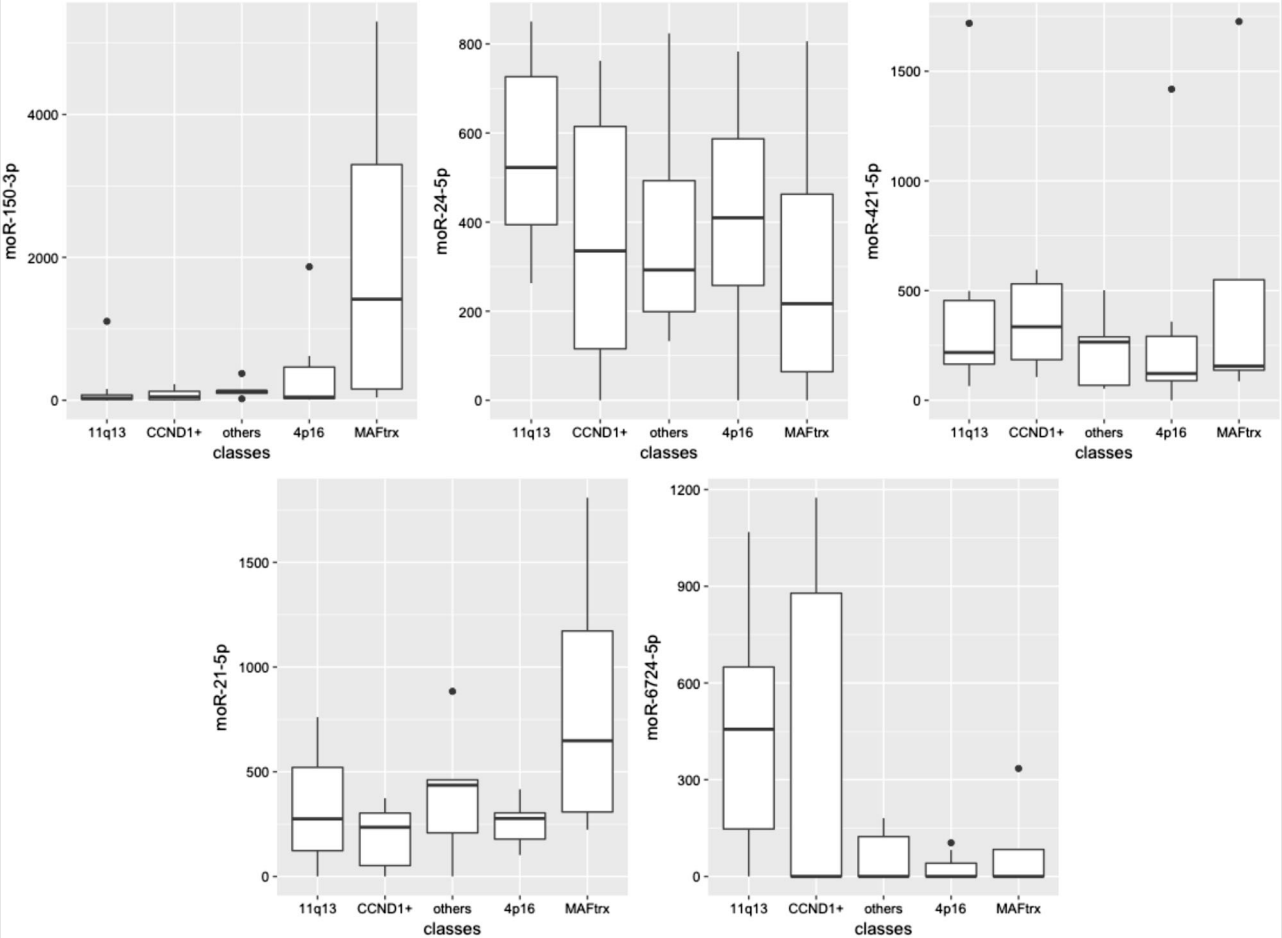
Expression of the most abundant moRNA in different molecular groups of MM patients, stratified on the basis of translocations/CCND1 classification

**Fig. 4:**
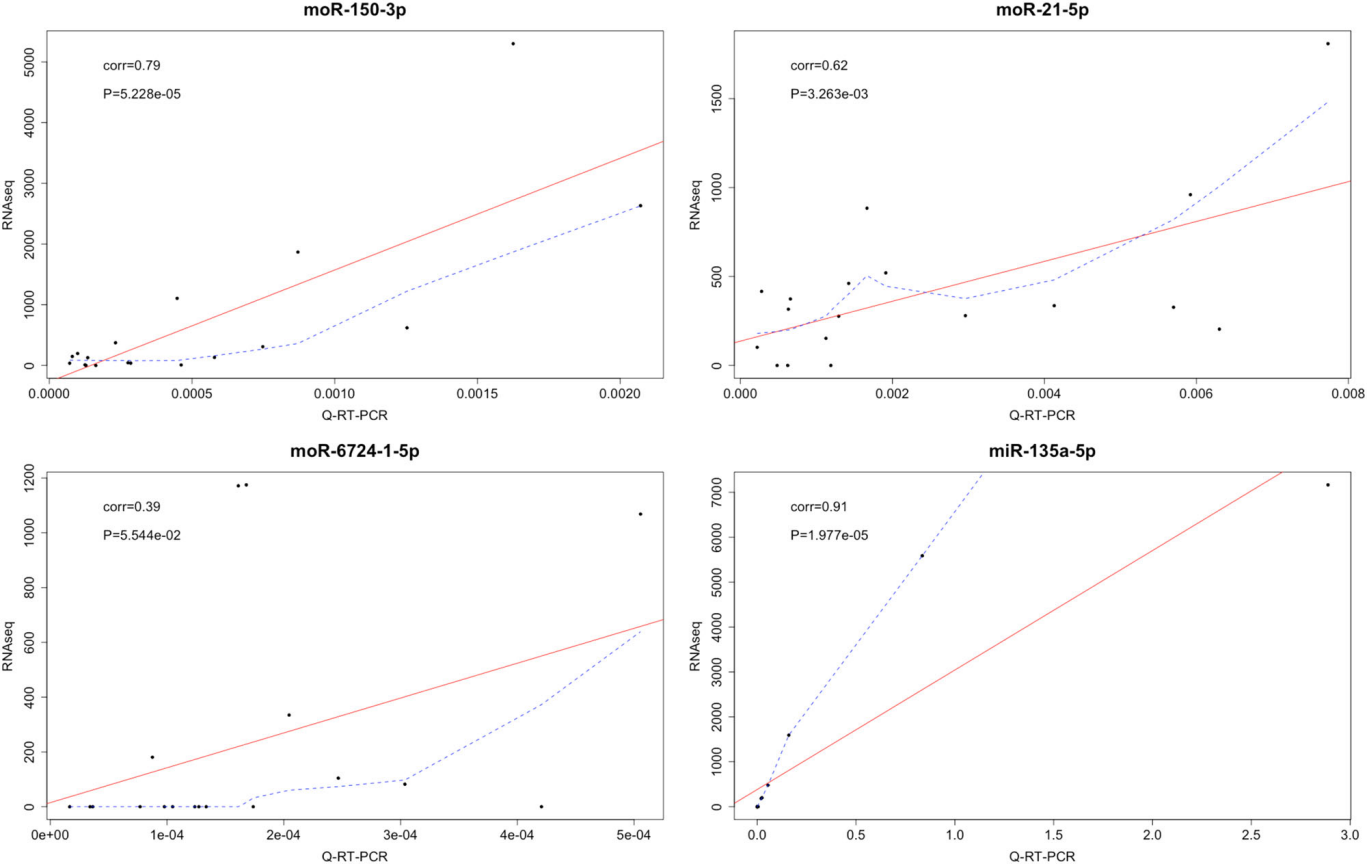
Validation by qRT-PCR for four sRNAs of differential expression detected by RNA-seq. Solid line: fitting linear regression; dashed line: fitting lowess regression

Among the highly expressed offset sRNAs, three were differentially expressed within the molecular subgroups: moR-150-3p and moR-21-5p in t(14;16)/t(14;20) samples, and moR-6724-1-5p in patients with high CCND1 levels (Fig. 3, all *P* *<* 1 × 10^−3^). qRT-PCR analysis of the expression level of miR-135-5p, moR-150-3p, moR-21-5p, and moR-6724-1-5p in a representative panel of cases for which RNA material was available and representing all the considered MM subgroups, confirmed the new findings and supported data reliability. Very highly significant correlations were found for the species analyzed (Fig. 4). Only for moR-6724-1-5p marginal significance was reached according to PCR estimations: in this case, we cannot exclude that discrepancies might be due to sequence length, in that the extended hairpin sequence, which possibly leads to moR-6724, could fold in a quite stable, non-conventional, hairpin configuration according to (predictions obtained by RNAfold (Supplementary Figure 4)).

The expression levels of the newly identified moRNAs were analyzed in a set of normal B-cell and PC populations from healthy donors. Notably, moR-6724-1-5p showed significantly high expression in non-pathological B- and PCs (Supplementary Figure 5), which prompts to suggest that its down-modulation could be somehow involved in the tumor cell development processes. A similar trend could be observed in moR-150 and moR-21 expression, where median levels in MM were overall lower, albeit not significantly, than in normal samples.

**Fig. 5:**
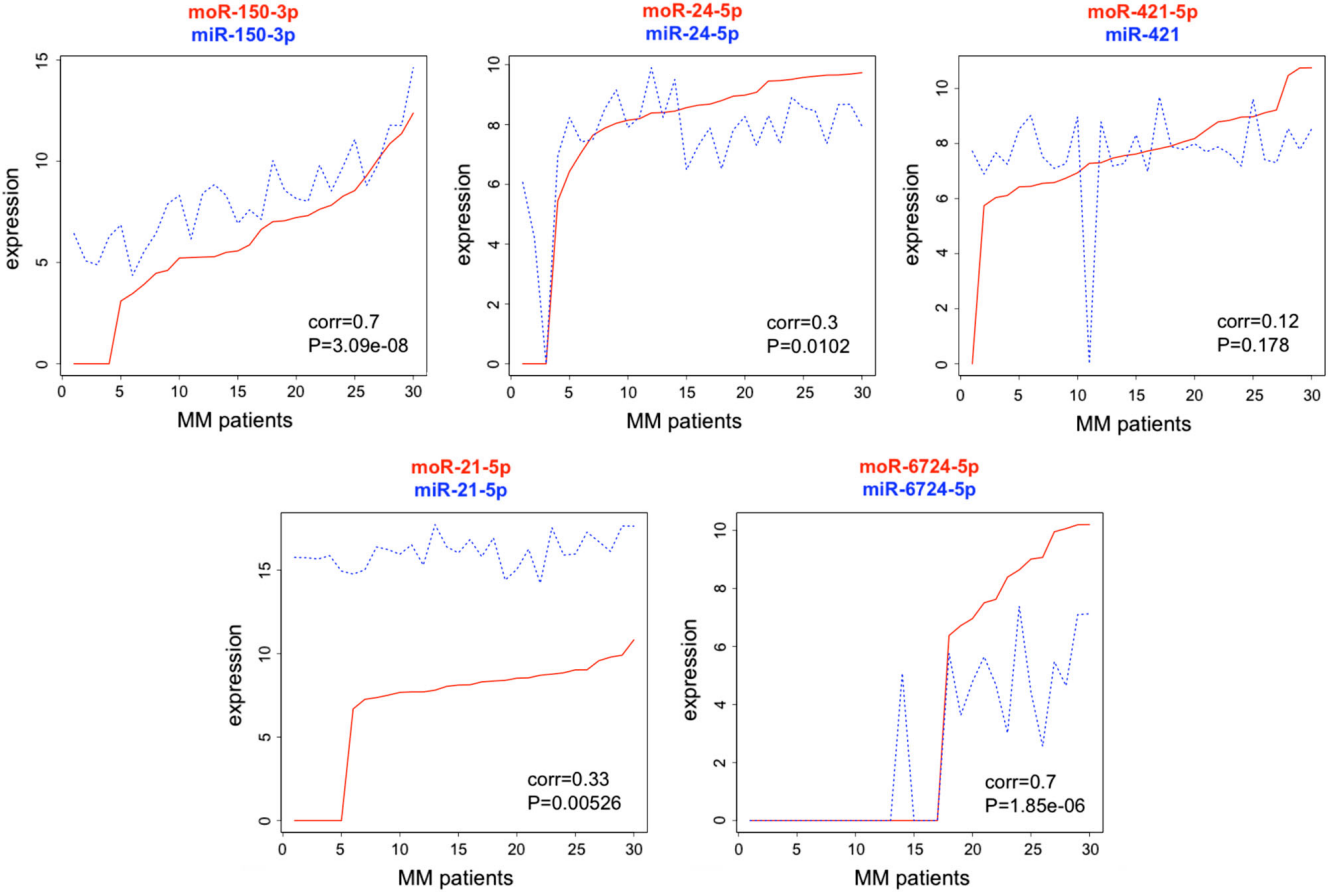
Correlation between expression profiles of selected moRNAs (solid lines) and the corresponding miRNA (dashed lines) derived from the same arm of the hairpin precursor. MM patients are sorted according to increasing moRNA expression level. Measure indicates Kendall’s tau correlation and significance for one-tailed test

In most cases, significant direct correlation was detected between the expression profiles of the most abundant moRNA and the miRNA derived from the same hairpin (Fig. 5), albeit with variable correlation strengths and ratios between the absolute levels of moRNA and its cognate miRNA. MoR-150-3p and moR-6724-5p profiles were highly correlated with the corresponding miRNAs, moR-6724-5p was more expressed than miR-6724-5p in a subset of samples. MoR-21-5p was considerably more weekly expressed than miR-21-5p and, notably, the moRNA was not expressed in a subset of samples presenting with high miR-21-5p levels. More divergent expression profiles were observed for moR-421-5p and miR-421-5p: both sRNAs showed variable expression in the considered set of cases, and none of them was differentially expressed in molecular subgroups.

### Prediction of putative moRNA transcriptional target in MM

To investigate the possible involvement of novel moRNA species in the regulation of MM transcriptional milieu, we first used in silico approach to extract a list of putative target transcripts based on the supposed high-affinity binding site. Although, to date, a clear demonstration has still not been provided, we might bona fide presume that the moRNA may act as conventional mature miRNAs to regulate possible targets target transcripts. Almost 800 coding and non-coding genes, which have been somehow related to MM to date according to the curated NCBI Gene annotation database, were considered to predict possible targets of the six most highly expressed moRNAs. The robust RNA22 target-prediction algorithm^34^ was used to extract and score predicted moRNA-target regulatory relationships (Supplementary Data). Amongst predicted targeting relationships (Supplementary Table 4), those involving moR-150-3p with *RUNX1* and moR-6724-1-5p with *TSC2* were of particular interest.

## Discussion

To date, the detection of miRNA transcripts in myeloma has been mainly confined to PCR- or array-based approaches. The high RNA-seq discovery power well balances the cost per sample, which sensibly decreased in the last years and is largely justified by the advantages of de novo investigations. Herein, we analyzed by RNA-seq a representative cohort of myeloma samples that had been previously characterized for molecular features and profiled by expression arrays, with the ultimate purpose of unraveling previously undescribed short sRNAs with potential implication in myeloma biology.

The first relevant finding concerned the relative abundance of known miRNA transcripts. Surprisingly, only four mature species seized up to about 50% of the reads, namely of the detected transcripts (additional six miRNAs increased the total capture level to 70%). Such miRNAs are commonly expressed at high level in several cell types, most likely contributing to physiological processes, and their overexpression is preferably associated with tumor suppressive activity. In PCs, miR-181a was described more expressed in MM than in healthy donors^36^. In our MM series, they were ubiquitously expressed independent of molecular subtypes. Circulating let-7a, miR-26a, and miR-181a were described as expressed at aberrant levels in tumor plasma or serum^37,38^.

Considering the finite set of miRNA contained in the arrays, RNA-seq data have reinforced previous findings. Moreover, RNA-seq data extended and completed the picture, surpassing the limitations of microarray analysis, affected by noise for the low-signal species and providing new discoveries. A marked upregulation of miR-135a-5p emerged in t(4;14) patients, not detected by microarray analysis neither in this nor in previous studies. Possibly, issues in array design and low specificity of array probes can explain this missing finding, since upregulation of miR-135a-5p in t(4;14) has been strongly confirmed by qRT-PCR in an extended cohort.

Abnormal expression of miR-135a has been documented in several cancer types, but the most striking evidence of the involvement of miR-135a in B-cell tumors was reported in Hodgkin’s lymphoma tumors, where Navarro et al.^39^ demonstrated the causal relationship that linked miR-135a to JAK2 downregulation and to the consequent disruption of STAT-mediated Bcl-xL anti-apoptotic function: low miR-135a level was significantly associated to higher proliferation rate and poorer outcome. Similarly, low mir-135a levels had also unfavorable, and independent, prognostic value in chronic lymphocytic leukemia^40^. Due to the limited number of cases in our series and the heterogeneous treatment received from patients with available follow-up (19 of 30), the prognostic significance of this miRNA in MM remains to be fully elucidated.

Importantly, RNA-seq analysis indicated several moRNA species expressed in myeloma. Most of the moRNAs stemmed from the 5’ arm of the hairpin, in close continuity if not overlapping to the so-called “exact” mature miRNA, in accordance with previous reports^21^. In support of this, the most reliable hypothesis, to date, suggests that moRNAs are secondary products of the Drosha/DGCR8-mediated processing of pre-miRNA^21^. As regards the validation of the obtained results, importantly, qRT-PCR analysis revealed high correlation with RNA-seq data, indicating the effectiveness of the detection of both annotated and newly detected sRNAs.

Little evidence have been published on the involvement of the offset RNA species in tumors and primarily limited to their structural description^15,20^ or to the identification of moRNAs differentially expressed in cancer cells^19^. In this regard, our report of differential moRNA expression in myeloma subgroups, as well as of their possible targeting relationships, encourages further investigation of the possible biological roles of these moRNAs.

Fundamental contributions to the interpretation of the moRNA functions, supporting the hypothesis that at least some of them can be post-transcriptional regulators of gene expression, were provided by recent studies. A first study on viral miRNAs and moRNAs expressed in virus-induced tumors of rhesus macaques gave, by luciferase reporter assay, the proof of principle that an artificial RNA containing a perfect target site for a moRNA can be targeted, and significantly downregulated, by the moRNA, confirming that a moRNA can act as a miRNA, but leaving open the question if actually moRNAs can effectively somehow regulate gene expression^15^. In this regard, two studies investigated by microarray analysis the effect on gene expression of the enforced expression of a moRNA. The first one, after reporting high and selective expression of moRNAs in embryonic stem cells, showed the gene expression modulation upon moR-103a-2-3p transfection, supporting the hypothesis that the moRNA acts directly as a post-transcriptional regulator^21^. Later, it has been demonstrated that moR-21 inhibits cell proliferation and mediates gene expression regulation in vascular smooth muscle cells^22^. Beyond consistently supporting the functionality of moRNAs, the two aforementioned studies provided somewhat discordant interpretations about a possible connection between moRNA and cognate miRNA roles. In fact, moR-103a-2-3p seemed to have functions that are not linked to the function of miR-103a-3p. Conversely, a close connection between miR-21 and moR-21 was outlined, showing that gene regulation was exerted by moR-21 in competitive way with miR-21, exploiting the presence in the target gene of either “seed” or “seed match” regions that allow modulation of gene expression. Overall, these findings sustain the notion of a close functional connection between different non-coding RNA species and prompt for further investigation aimed at considering a comprehensive portrait of these sequences in MM.

In conclusion, RNA-seq of a representative panel of MM tumors confirmed and extended miRNA transcriptional profiling data obtained by microarray, and generated a more complete overview of small non-coding RNA in MM, unraveling also specific moRNAs that demand further investigations and may represent, like miRNAs, primary actors in MM biology.

## Supplementary information

Supplementary Figures.

Supplementary Tables 1-4.

Supplementary Data.

## Data Availability

The whole R code used to generate raw small ncRNA data is freely available upon request to the Authors.

## Data availability

The RNA-seq raw data are publicly available in GEO repository under accession #GSE117365. The microarray raw and normalized data are publicly available in GEO repository under accession #GSE117113.
