# Supplementary material for: Expanding the repertoire of miRNAs and miRNA-offset RNAs expressed in multiple myeloma by small RNA deep sequencing
Source: Blood Cancer J. 2019 Feb 19;9(3):21. doi: 10.1038/s41408-019-0184-x (PMC6381125; doi:10.1038/s41408-019-0184-x)
Supplement: Supplementary file 1 — Supplementary Figures. [file 41408_2019_184_MOESM1_ESM.pdf]

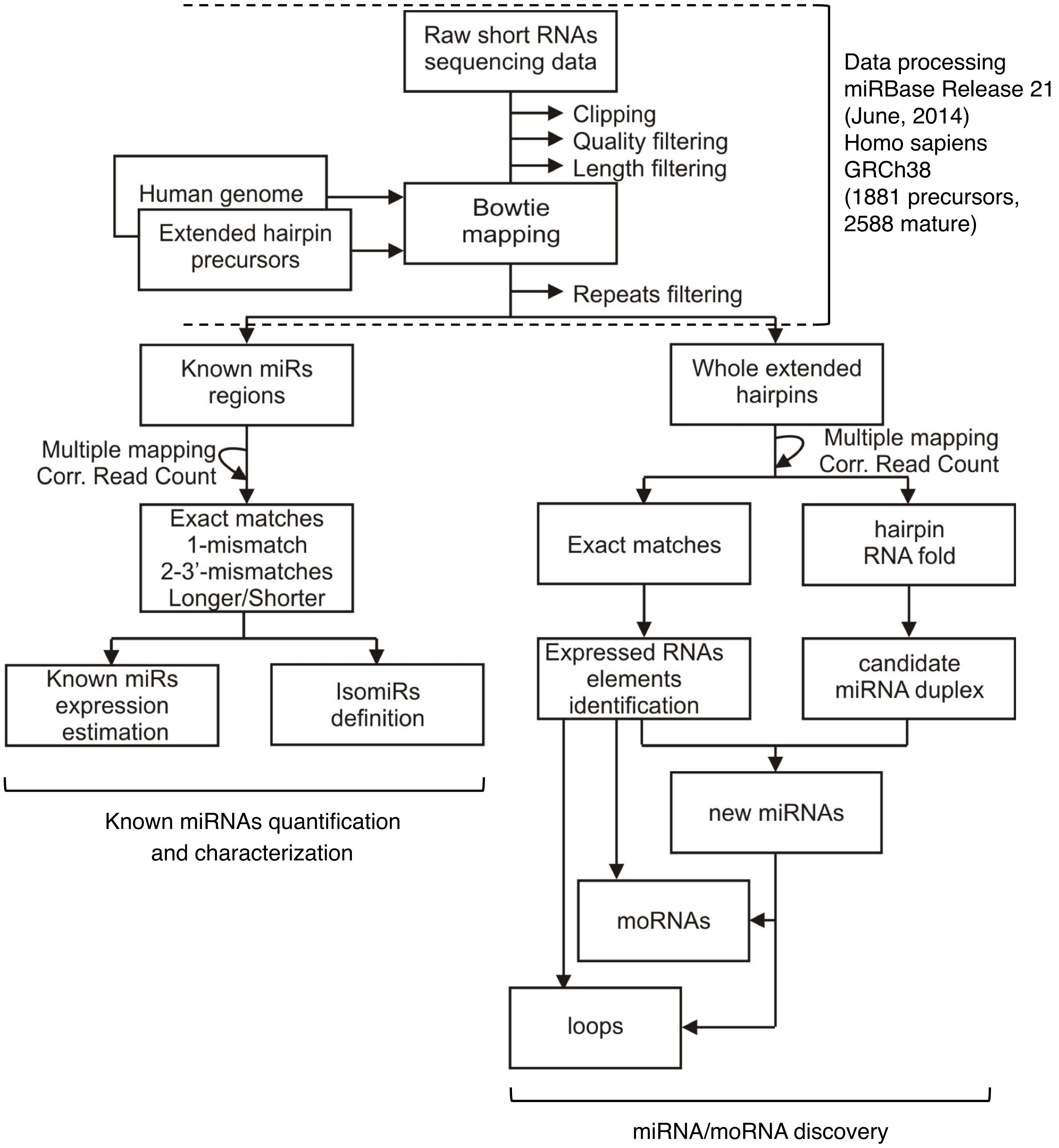

Supplementary Figure 1. small RNAseq analysis workflow.

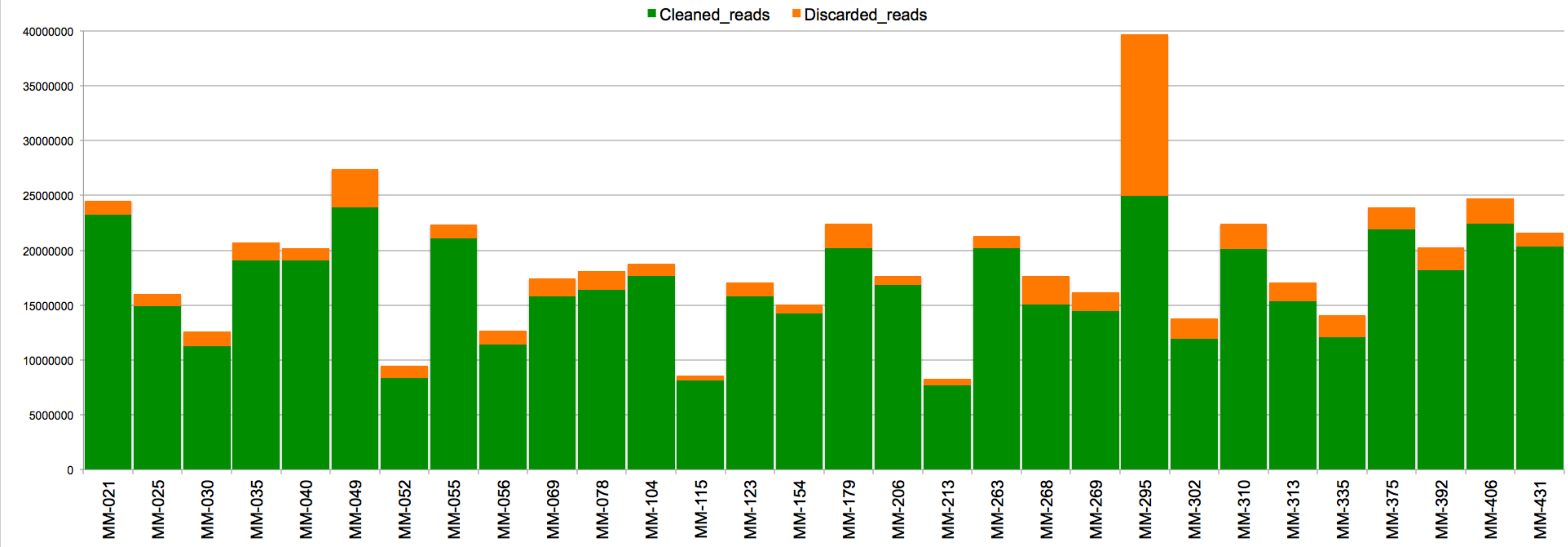

	MM-021	MM-025	MM-030	MM-035	MM-040	MM-049	MM-052	MM-055	MM-056	MM-069	MM-078	MM-104	MM-115	MM-123	MM-154	MM-179	MM-206	MM-213	MM-263	MM-268	MM-269	MM-295	MM-302	MM-310	MM-313	MM-335	MM-375	MM-392	MM-406	MM-431
Raw reads	24.5	16.0	12.6	20.7	20.2	27.4	9.5	22.3	12.7	17.4	18.1	18.8	8.6	17.1	15.0	22.4	17.6	8.2	21.3	17.7	16.2	39.7	13.8	22.4	17.1	14.1	23.9	20.3	24.8	21.6
Discarded reads	1.3	1.2	1.4	1.7	1.1	3.5	1.1	1.2	1.3	1.6	1.7	1.1	0.4	1.3	0.8	2.2	0.8	0.6	1.1	2.6	1.7	14.7	1.8	2.3	1.8	2.0	2.0	2.1	2.3	1.3
Cleaned reads	23.2	14.9	11.2	19.1	19.1	23.9	8.4	21.1	11.4	15.8	16.4	17.7	8.1	15.8	14.2	20.2	16.9	7.7	20.2	15.1	14.5	25.0	11.9	20.1	15.3	12.1	21.9	18.2	22.4	20.3

Supplementary Figure 2. Histogram of raw reads count in the 30 MM samples. Raw, discarded and cleaned million reads are reported below.

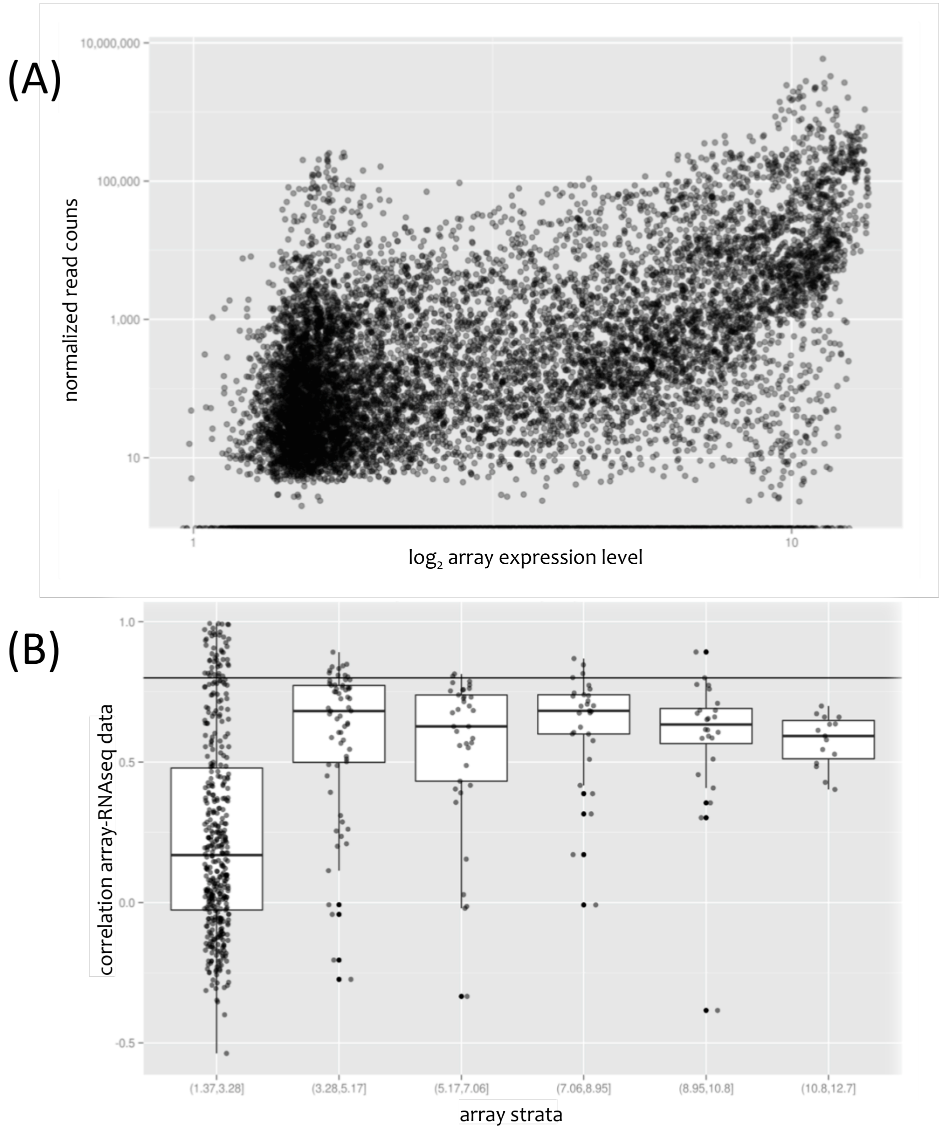

Supplementary Figure 3. (A) Scatter plot showing the relationship between expression data of each transcript for each sample as evaluated by arrays (x-axis) or RNAseq (y-axis). The fraction of those transcript with high array detection that did not present any read represented the 0.44% of the total signals; less than 0.015% of the total signals had array logged expression higher than 2^6. **(B)** Box plot and strip plot dispersion of the linear correlation coefficients of the relationship between array- and RNA-seq-based miRNA expression levels, stratified according to array expression (five equally spaced thresholds).

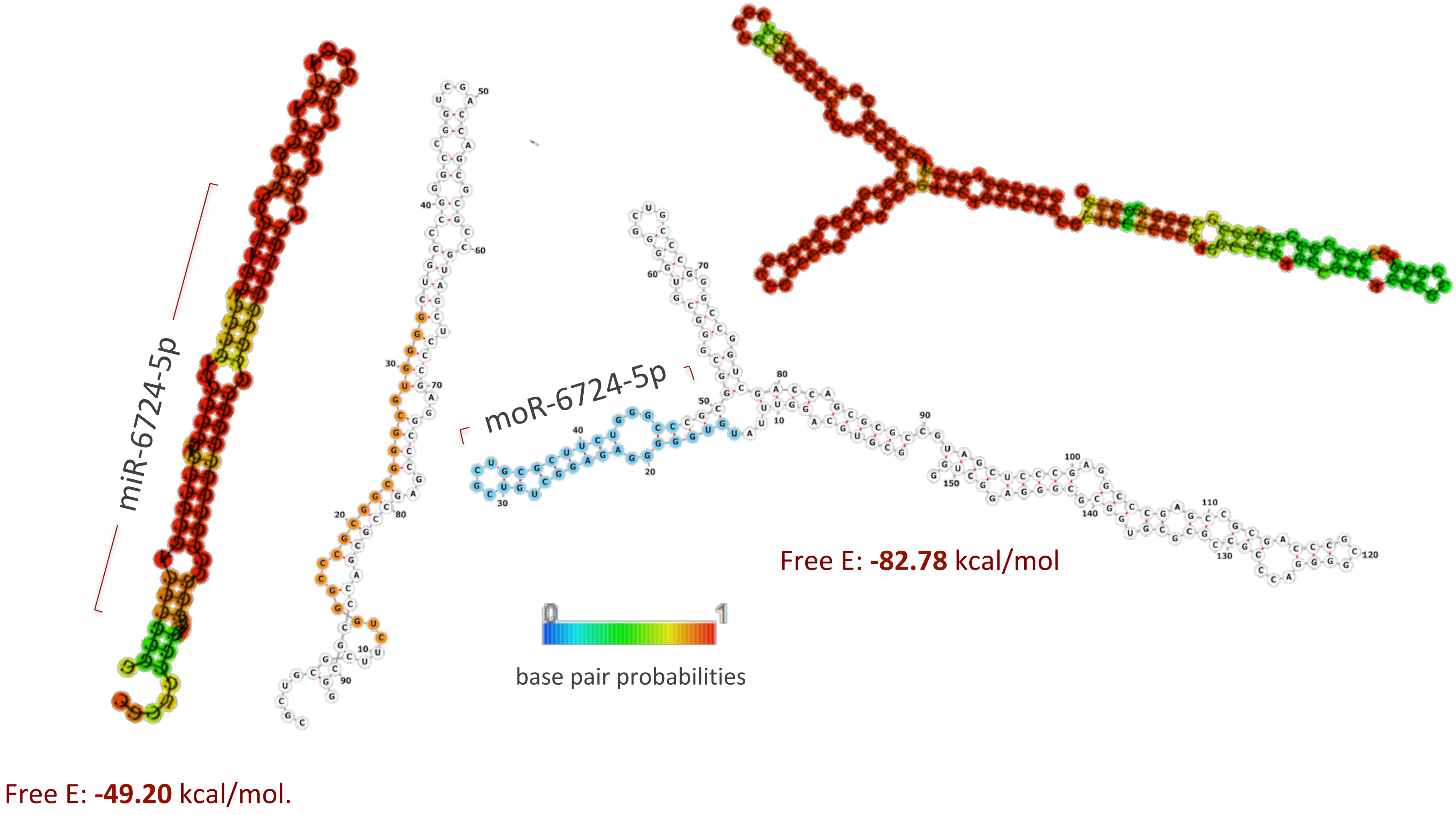

Supplementary Figure 4. RNAfold analysis of conventional and extended mir-6274 hairpins, showing the positions of miR-6724-5p and moR-6724-5p sequences.

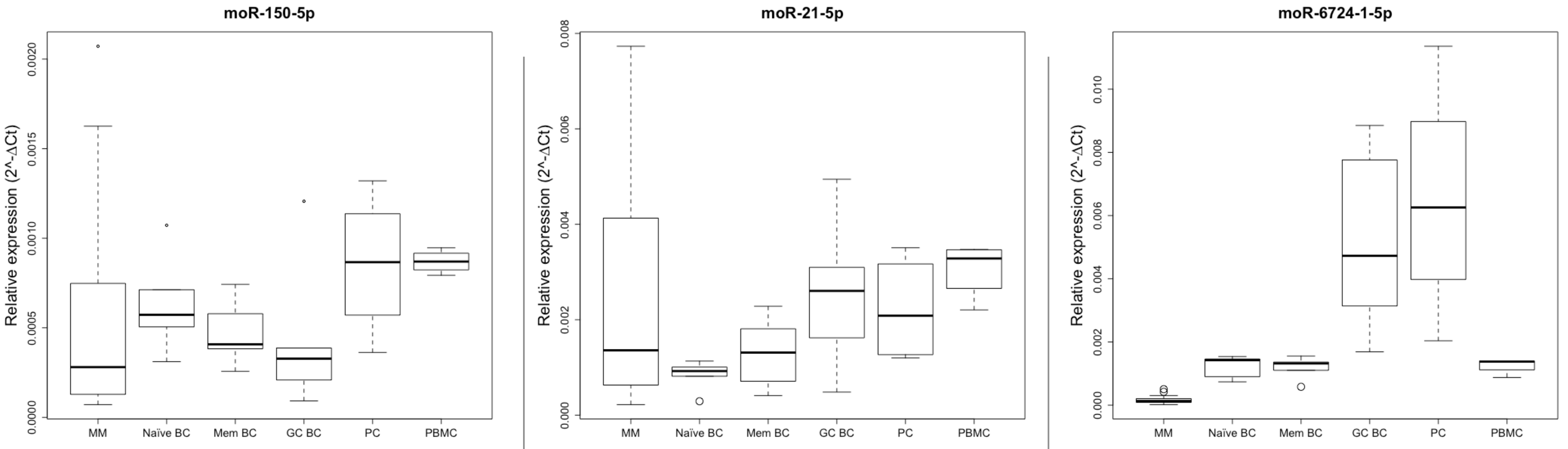


```
Kruskal-Wallis chi-squared = 8.3216, df = 5, p-value = 0.14


                           Comparison of x by g
                            (Benjamini-Hochberg)
Col Mean-|
Row Mean |      GC BC   Memory B         MM   Naïve BC       PBMC
---------+-------------------------------------------------------
Memory B |  -0.435589
         |     0.4145
         |
      MM |  -0.158717   0.386242
         |     0.4369     0.4035
         |
Naïve BC |  -1.029574  -0.593985  -1.129368
         |     0.2843     0.3767     0.2772
         |
    PBMC |  -1.941383  -1.530706  -2.210833  -0.970691
         |     0.1958     0.1888     0.2029     0.2764
         |
      PC |  -1.661376  -1.250698  -1.871027  -0.690684   0.265638
         |     0.1812     0.2638     0.1534     0.3673     0.4235
```

```
Kruskal-Wallis chi-squared = 8.1573, df = 5, p-value = 0.15


                           Comparison of x by g
                            (Benjamini-Hochberg)
Col Mean-|
Row Mean |      GC BC   Memory B         MM   Naïve BC       PBMC
---------+-------------------------------------------------------
Memory B |   1.042773
         |     0.2785
         |
      MM |   0.652300  -0.652300
         |     0.2755     0.2967
         |
Naïve BC |   1.834753   0.791980   1.643135
         |     0.2495     0.3213     0.1505
         |
    PBMC |  -0.815132  -1.798268  -1.585758  -2.544954
         |     0.3458     0.1353     0.1410     0.0820
         |
      PC |  -0.068446  -1.051582  -0.679610  -1.798268   0.708368
         |     0.4727     0.3139     0.3105     0.1803     0.3264
```

```
Kruskal-Wallis chi-squared = 34.6867, df = 5, p-value = 0


                           Comparison of x by g
                            (Benjamini-Hochberg)
Col Mean-|
Row Mean |      GC BC   Memory B         MM   Naïve BC       PBMC
---------+-------------------------------------------------------
Memory B |   1.570760
         |     0.1245
         |
      MM |   4.475273   2.510116
         |    0.0001*    0.0226*
         |
Naïve BC |   1.346366  -0.224394  -2.790853
         |     0.1215     0.5140    0.0131*
         |
    PBMC |   1.396924  -0.084002  -2.397516   0.127558
         |     0.1354     0.4665    0.0248*     0.5184
         |
      PC |  -0.112002  -1.592929  -4.228690  -1.381368  -1.431494
         |     0.4879     0.1390    0.0001*     0.1254     0.1428
```

Supplementary Figure 5. Boxplot of moR-150-5p, moR-21-5p and moR-6724-1-5p expression levels assessed by Q-RT-PCR in multiple myeloma (MM) patients; in plasma cells (PC), germinal center B-cells, Naïve B-cells, and memory B-cells (Mem BC) samples from tonsil avulsion during standard surgery; and in whole peripheral blood mononucleated cells (PBMC) from peripheral blood samples. Kruskall Wallis test and Dunn's test comparison (with Benjamini and Hochberg multiple test correction) metrics are reported below.
