# Supplementary material for: Expanding the repertoire of miRNAs and miRNA-offset RNAs expressed in multiple myeloma by small RNA deep sequencing
Source: Blood Cancer J. 2019 Feb 19;9(3):21. doi: 10.1038/s41408-019-0184-x (PMC6381125; doi:10.1038/s41408-019-0184-x)
Supplement: Supplementary file 2 — Supplementary Tables 1-4. [file 41408_2019_184_MOESM2_ESM.pdf]

Supplementary Table 1. Patients' molecular features. The "group" categorical variable was defined according to the IGH Translocations/CyclinsD-based classification. Briefly, "11q13" were characterized by the t(11;14) with the consequent over-expression of CCND1, and a nonhyperdiploid status; "CCND1+" showed low to moderate levels of the CCND1 gene in the absence of any primary IGH translocation and is mostly - but not exclusively - associated with a hyperdiploid status; "4p16" showed high CCND2 levels and the presence of the t(4;14) translocation; "MAFtrx" expressed the highest levels of CCND2 in association with either the t(14;16) or t(14;20) translocations, deregulating MAF-family genes members; and finally "other" included tumors that do not fall into any of the other groups, albeit most of which expressing CCND2. HD: hyperdiploidy; "+" indicates genetic alterations or DIS3, N-/K-RAS, BRAF3 mutations occurrence; NA: not available.

Pts	group	del13	del17p	gain 1q	HD	DIS3	NRAS	KRAS	BRAF3
MM.021	4p16	+	+	+	-	NA	NA	NA	NA
MM.025	MAFtrx	+	-	+	-	NA	NA	NA	NA
MM.030	CCND1+	-	-	-	+	-	-	+	-
MM.035	CCND1+	-	-	-	-	NA	NA	NA	NA
MM.040	other	+	-	+	+	NA	NA	NA	NA
MM.049	CCND1+	-	-	-	+	-	+	-	-
MM.052	11q13	+	-	+	-	NA	NA	NA	NA
MM.055	11q13	-	-	-	-	+	-	+	-
MM.056	CCND1+	-	-	NA	-	NA	NA	NA	NA
MM.069	MAFtrx	+	-	+	-	-	-	-	-
MM.078	other	+	-	-	+	-	-	-	-
MM.104	4p16	+	-	+	-	NA	NA	NA	NA
MM.115	11q13	+	-	+	-	-	-	+	-
MM.123	4p16	+	-	+	-	+	-	-	-
MM.154	MAFtrx	+	-	+	-	-	-	+	-
MM.179	11q13	-	+	-	-	-	-	-	-
MM.206	4p16	+	-	-	-	-	-	-	-
MM.213	11q13	+	-	-	-	+	-	-	-
MM.263	4p16	+	-	+	-	+	-	-	-
MM.268	CCND1+	-	-	-	-	-	-	-	+
MM.269	other	+	-	-	+	-	-	+	-
MM.295	other	+	-	NA	-	-	+	-	+
MM.302	CCND1+	-	-	+	+	-	+	-	-
MM.310	11q13	-	-	-	-	+	-	+	-
MM.313	11q13	-	-	-	-	-	-	-	+
MM.335	MAFtrx	+	-	+	-	+	-	+	+
MM.375	4p16	-	+	-	-	-	-	-	-
MM.392	other	+	-	+	+	-	-	-	-
MM.406	11q13	-	-	+	-	-	-	-	-
MM.431	4p16	+	-	+	-	-	-	-	-

Supplementary Table 2. Normalized read counts of the 17 newly detected mature miRNAs.

	MM. 055	MM. 115	MM. 213	MM. 310	MM. 179	MM. 406	MM. 313	MM. 052	MM. 030	MM. 035	MM. 049	MM. 302	MM. 056	MM. 268	MM. 078	MM. 269	MM. 295	MM. 392	MM. 040	MM. 021	MM. 123	MM. 375	MM. 431	MM. 104	MM. 206	MM. 263	MM. 069	MM. 154	MM. 335	MM. 025	max value	Sum of reads
**hsa-miR-1303***	54.66	76.07	0.00	38.57	0.00	25.99	26.66	74.09	55.43	89.58	12.57	88.22	16.80	56.35	41.82	191.90	63.73	107.30	139.75	57.07	108.97	0.00	17.44	22.67	43.07	156.50	95.94	20.73	198.50	0.00	**198.50**	**1880.36**
**hsa-miR-135a-2-3p**	0.00	0.00	0.00	0.00	0.00	0.00	0.00	0.00	0.00	0.00	0.00	0.00	0.00	0.00	0.00	0.00	0.00	112.62	0.00	11.41	0.00	0.00	653.28	309.12	19.48	19.39	0.00	0.00	0.00	0.00	**653.28**	**1125.31**
**hsa-miR-5696***	28.25	19.50	48.04	29.71	30.20	25.99	39.99	39.63	0.00	43.94	49.37	62.55	15.27	60.69	82.67	28.58	13.66	19.51	14.08	25.87	35.19	0.00	21.50	37.03	16.41	30.76	21.89	20.73	44.02	0.00	**82.67**	**905.05**
**hsa-miR-4424***	0.00	0.00	0.00	6.32	0.00	22.56	0.00	0.00	0.00	0.00	0.00	0.00	0.00	0.00	38.90	0.00	18.21	12.41	179.96	15.22	12.49	0.00	312.69	20.41	0.00	17.39	0.00	8.77	0.00	0.00	**312.69**	**665.33**
**hsa-miR-2110***	6.14	0.00	0.00	6.95	0.00	0.00	0.00	0.00	0.00	0.00	0.00	0.00	0.00	0.00	11.67	0.00	0.00	0.00	12.06	24.35	51.08	10.98	7.56	8.31	0.00	46.82	9.66	13.55	22.41	402.26	**402.26**	**633.81**
**hsa-miR-4473***	118.54	23.40	0.00	24.66	54.24	21.58	27.69	0.00	0.00	0.00	0.00	0.00	16.80	14.45	14.59	12.25	11.38	15.96	0.00	0.00	26.11	0.00	23.25	0.00	0.00	10.03	9.01	8.77	24.81	0.00	**118.54**	**457.52**
**hsa-miR-153-1-5p**	0.00	0.00	28.02	0.00	5.59	0.00	0.00	0.00	0.00	0.00	0.00	0.00	0.00	0.00	0.00	0.00	0.00	0.00	0.00	0.00	55.62	0.00	7.56	0.00	0.00	12.71	0.00	0.00	40.82	55.10	**55.62**	**205.42**
**hsa-miR-650***	0.00	0.00	130.10	0.00	0.00	0.00	0.00	0.00	0.00	0.00	0.00	0.00	0.00	0.00	0.00	0.00	0.00	0.00	0.00	0.00	0.00	0.00	0.00	0.00	0.00	0.00	0.00	0.00	0.00	0.00	**130.10**	**130.10**
**hsa-miR-4487***	0.00	0.00	0.00	8.85	0.00	0.00	0.00	0.00	19.00	0.00	9.87	19.25	0.00	21.67	0.00	0.00	6.37	0.00	0.00	0.00	0.00	0.00	0.00	0.00	0.00	0.00	0.00	0.00	0.00	0.00	**21.67**	**85.02**
**hsa-miR-421***	0.00	0.00	0.00	6.32	0.00	0.00	0.00	0.00	0.00	0.00	0.00	0.00	0.00	0.00	0.00	0.00	5.92	0.00	0.00	0.00	0.00	0.00	11.62	0.00	0.00	0.00	7.08	8.77	17.61	0.00	**17.61**	**57.33**
**hsa-miR-548ag-2***	0.00	0.00	0.00	0.00	0.00	0.00	0.00	0.00	0.00	0.00	0.00	0.00	0.00	0.00	0.00	0.00	0.00	0.00	25.13	0.00	0.00	0.00	13.95	0.00	0.00	0.00	0.00	0.00	0.00	0.00	**25.13**	**39.08**
**hsa-miR-1269a***	0.00	0.00	0.00	0.00	0.00	0.00	0.00	0.00	0.00	0.00	0.00	0.00	0.00	0.00	0.00	0.00	0.00	0.00	0.00	0.00	0.00	0.00	0.00	0.00	0.00	0.00	0.00	0.00	31.22	0.00	**31.22**	**31.22**
**hsa-miR-600***	0.00	0.00	0.00	0.00	0.00	17.16	0.00	0.00	0.00	0.00	0.00	0.00	0.00	0.00	0.00	0.00	0.00	0.00	0.00	0.00	0.00	4.77	0.00	0.00	0.00	0.00	0.00	0.00	0.00	0.00	**17.16**	**21.94**
**hsa-miR-137***	17.20	0.00	0.00	0.00	0.00	0.00	0.00	0.00	0.00	0.00	0.00	0.00	0.00	0.00	0.00	0.00	0.00	0.00	0.00	0.00	0.00	0.00	0.00	0.00	0.00	0.00	0.00	0.00	0.00	0.00	**17.20**	**17.20**
**hsa-miR-466***	0.00	0.00	0.00	0.00	0.00	0.00	0.00	0.00	0.00	0.00	0.00	0.00	0.00	0.00	0.00	0.00	0.00	0.00	0.00	0.00	0.00	0.00	0.00	0.00	0.00	0.00	0.00	0.00	12.81	0.00	**12.81**	**12.81**
**hsa-miR-549a***	0.00	0.00	0.00	0.00	0.00	0.00	0.00	0.00	0.00	0.00	0.00	0.00	0.00	0.00	0.00	0.00	0.00	0.00	0.00	0.00	0.00	0.00	0.00	0.00	0.00	0.00	0.00	9.57	0.00	0.00	**9.57**	**9.57**
**hsa-miR-5682***	0.00	0.00	0.00	0.00	0.00	5.88	0.00	0.00	0.00	0.00	0.00	0.00	0.00	0.00	0.00	0.00	0.00	0.00	0.00	0.00	0.00	0.00	0.00	0.00	0.00	0.00	0.00	0.00	0.00	0.00	**5.88**	**5.88**

Supplementary Table 3. To assess whether the discrepancies between microarray and RNAseq technologies could affect the detection of true biological effects (apart from what observed for values just above the background), we evaluated the percentage of overlapping results in the detection of differentially expressed known miRNAs between prognostic groups, for convenience according to translocation/cyclinD classification [23,33]. Each of the five groups was compared, in coupled RNA-seq and microarray dataset, with all the remaining cases to find significantly associated transcripts, either in RNA-seq or in array data. Table reports the absolute number of reads (and percentages) of intersections between differential expression analyses. Full intersection considers transcript overlapping independent of modulation direction. Means of percentanges and of percentage ratios (last raw) are reported in the last column. Specifically, on average, absolute 71.05% (range: 63.89%-77.14%) of the miRNAs detected in the RNA-seq datasets were concordantly detected in microarray data. Such a percentage raised to 79.7% if concordance was evaluated on the intersection of detected transcripts by the two methods); conversely, absolute 66.51% (range 35%-90.16%) of the differentially expressed miRNAs resulting from microarray analysis were concordantly detected by RNA-seq (92.7% of those commonly detected).

RNA-seq data detected by arrays	**11q13**	**CCND1+**	**other**	**4p16**	**MAFtrx**	**% mean**
full intersection (A)	33 (94.29%)	28 (90.32%)	31 (86.11%)	47 (92.16%)	39 (82.98%)	89.17%
concordant data (B)	27 (77.14%)	22 (70.97%)	23 (63.89%)	37 (72.55%)	33 (70.72%)	71.05%
relative percentages (A/B)	81.81%	78.57%	74.2%	78.72%	85.23%	79.7%

Array data detected by RNA-seq	**11q13**	**CCND1+**	**other**	**4p16**	**MAFtrx**	**% mean**
full intersection	33 (86.84%)	58 (95.08%)	6 (54.55%)	18 (45%)	17 (73.91%)	71.08%
concordant data	30 (78.95%)	55 (90.16%)	6 (54.55%)	14 (35%)	17 (73.91%)	66.51%
relative percentage	90.91%	94.82%	100%	77.78%	100%	92.7%

Supplementary Table 4. Output of significant relationship (q<0.05) in RNA22 analysis. Deatiled fields description is available at https://cm.jefferson.edu/data-tools-downloads/rna22-full-sets-of-predictions/

<table>
<tr><th></th><th>moRNA</th><th>transcript</th><th>target.position</th><th>E.kcal.mol</th><th>target.site.seq</th><th>moR.seq</th><th>heteroduplex.pairing.1</th><th>heteroduplex.pairing.2</th><th>number.of.pairings</th><th>span.pred.target</th><th>p.val</th><th>q.val</th></tr>
<tr><td>**moR-6724-1-5p**</td><td>ENST00000628768.1|ENSG00000136854.20|OTTHUMG00000020713.12|OTTHUMT00000481731.1|STXBP1-212|STXBP1|2582|processed_transcript|</td><td>target</td><td>from/to=[634,666]</td><td>-22.9</td><td>TCCTCCAGGATCCACACACATCCTCACCCCACA</td><td>TGTGGGGGAGAGGCTGTCGCTGCGCTTCTGGGCC</td><td>................(((.((((.(((((((</td><td>)))))))..)))).)))................</td><td>14</td><td>33</td><td>0</td><td>0</td></tr>
<tr><td>**moR-6724-1-5p**</td><td>ENST00000530688.5|ENSG00000177685.16|OTTHUMG00000165267.7|OTTHUMT00000383098.1|CRACR2B-210|CRACR2B|1623|retained_intron|</td><td>target</td><td>from/to=[586,621]</td><td>-28.1</td><td>GGGAGGTGAGCACCCGGCCCCTGCCCTGTCCCCACG</td><td>TGTGGGGGAGAGGCTGTCGCTGCGCTTCTGGGCC</td><td>.......((((...((((..(.(((((.((((((((</td><td>)))))))))).))).)..)))).)))).......</td><td>22</td><td>36</td><td>2.00E-06</td><td>0.014142</td></tr>
<tr><td>**moR-6724-1-5p**</td><td>ENST00000528694.1|ENSG00000177685.16|OTTHUMG00000165267.7|OTTHUMT00000383100.1|CRACR2B-208|CRACR2B|2440|retained_intron|</td><td>target</td><td>from/to=[1403,1438]</td><td>-28.1</td><td>GGGAGGTGAGCACCCGGCCCCTGCCCTGTCCCCACG</td><td>TGTGGGGGAGAGGCTGTCGCTGCGCTTCTGGGCC</td><td>.......((((...((((..(.(((((.((((((((</td><td>)))))))))).))).)..)))).)))).......</td><td>22</td><td>36</td><td>2.00E-06</td><td>0.014142</td></tr>
<tr><td>**moR-6724-1-5p**</td><td>ENST00000643088.1|ENSG00000103197.17|OTTHUMG00000128745.21|OTTHUMT00000495035.1|TSC2-235|TSC2|5970|protein_coding|</td><td>target</td><td>from/to=[5611,5640]</td><td>-21.5</td><td>CACACACACACACAGTCACCTTCCTCCACC</td><td>TGTGGGGGAGAGGCTGTCGCTGCGCTTCTGGGCC</td><td>............((((((((((((((((.</td><td>.))))))))).))).))..))))............</td><td>17</td><td>30</td><td>0</td><td>0</td></tr>
<tr><td>**moR-6724-1-5p**</td><td>ENST00000643946.1|ENSG00000103197.17|OTTHUMG00000128745.21|OTTHUMT00000495031.2|TSC2-243|TSC2|6344|protein_coding|</td><td>target</td><td>from/to=[5743,5772]</td><td>-21.5</td><td>CACACACACACACAGTCACCTTCCTCCACC</td><td>TGTGGGGGAGAGGCTGTCGCTGCGCTTCTGGGCC</td><td>............((((((((((((((((.</td><td>.))))))))).))).))..))))............</td><td>17</td><td>30</td><td>0</td><td>0</td></tr>
<tr><td>**moR-6724-1-5p**</td><td>ENST00000642791.1|ENSG00000103197.17|OTTHUMG00000128745.21|OTTHUMT00000495068.2|TSC2-231|TSC2|1855|retained_intron|</td><td>target</td><td>from/to=[1297,1326]</td><td>-21.5</td><td>CACACACACACACAGTCACCTTCCTCCACC</td><td>TGTGGGGGAGAGGCTGTCGCTGCGCTTCTGGGCC</td><td>............((((((((((((((((.</td><td>.))))))))).))).))..))))............</td><td>17</td><td>30</td><td>0</td><td>0</td></tr>
<tr><td>**moR-150-3p**</td><td>ENST00000344691.8|ENSG00000159216.18|OTTHUMG00000086299.10|OTTHUMT00000194230.1|RUNX1-202|RUNX1|7274|protein_coding|</td><td>target</td><td>from/to=[5412,5438]</td><td>-20.4</td><td>ACTGTAGTTGTTTTGGCGATAGGTCTC</td><td>GGGACCTGGGGACCCCGGCACCGGCAGG</td><td>.((((.(.((((..(((..((((((((</td><td>))))))))..).))..)))).).)))).</td><td>20</td><td>27</td><td>4.00E-06</td><td>0.028268</td></tr>
<tr><td>**moR-150-3p**</td><td>ENST00000437180.5|ENSG00000159216.18|OTTHUMG00000086299.10|-|RUNX1-207|RUNX1|5967|protein_coding|</td><td>target</td><td>from/to=[4105,4131]</td><td>-20.4</td><td>ACTGTAGTTGTTTTGGCGATAGGTCTC</td><td>GGGACCTGGGGACCCCGGCACCGGCAGG</td><td>.((((.(.((((..(((..((((((((</td><td>))))))))..).))..)))).).)))).</td><td>20</td><td>27</td><td>4.00E-06</td><td>0.028268</td></tr>
<tr><td>**moR-150-3p**</td><td>ENST00000300305.7|ENSG00000159216.18|OTTHUMG00000086299.10|OTTHUMT00000194231.1|RUNX1-201|RUNX1|6222|protein_coding|</td><td>target</td><td>from/to=[4360,4386]</td><td>-20.4</td><td>ACTGTAGTTGTTTTGGCGATAGGTCTC</td><td>GGGACCTGGGGACCCCGGCACCGGCAGG</td><td>.((((.(.((((..(((..((((((((</td><td>))))))))..).))..)))).).)))).</td><td>20</td><td>27</td><td>3.00E-06</td><td>0.021207</td></tr>
<tr><td>**moR-150-3p**</td><td>ENST00000344691.8|ENSG00000159216.18|OTTHUMG00000086299.10|OTTHUMT00000194230.1|RUNX1-202|RUNX1|7274|protein_coding|</td><td>target</td><td>from/to=[5412,5438]</td><td>-20.4</td><td>ACTGTAGTTGTTTTGGCGATAGGTCTC</td><td>GGGACCTGGGGACCCCGGCACCGGCAGG</td><td>.((((.(.((((..(((..((((((((</td><td>))))))))..).))..)))).).)))).</td><td>20</td><td>27</td><td>4.00E-06</td><td>0.028268</td></tr>
<tr><td>**moR-150-3p**</td><td>ENST00000437180.5|ENSG00000159216.18|OTTHUMG00000086299.10|-|RUNX1-207|RUNX1|5967|protein_coding|</td><td>target</td><td>from/to=[4105,4131]</td><td>-20.4</td><td>ACTGTAGTTGTTTTGGCGATAGGTCTC</td><td>GGGACCTGGGGACCCCGGCACCGGCAGG</td><td>.((((.(.((((..(((..((((((((</td><td>))))))))..).))..)))).).)))).</td><td>20</td><td>27</td><td>4.00E-06</td><td>0.028268</td></tr>
<tr><td>**moR-150-3p**</td><td>ENST00000300305.7|ENSG00000159216.18|OTTHUMG00000086299.10|OTTHUMT00000194231.1|RUNX1-201|RUNX1|6222|protein_coding|</td><td>target</td><td>from/to=[4360,4386]</td><td>-20.4</td><td>ACTGTAGTTGTTTTGGCGATAGGTCTC</td><td>GGGACCTGGGGACCCCGGCACCGGCAGG</td><td>.((((.(.((((..(((..((((((((</td><td>))))))))..).))..)))).).)))).</td><td>20</td><td>27</td><td>3.00E-06</td><td>0.021207</td></tr>
</table>
